# Nonlinear electrocardiographic imaging using polynomial approximation networks

**DOI:** 10.1063/1.5038046

**Published:** 2018-10-16

**Authors:** Abhejit Rajagopal, Vincent Radzicki, Hua Lee, Shivkumar Chandrasekaran

**Affiliations:** Department of Electrical and Computer Engineering, University of California, Santa Barbara, California 93106, USA

## Abstract

Electrocardiography is a valuable tool to aid in medical understanding and treatment of heart-related ailments, specifically atrial fibrillation (AF) and other irregular cardiac behavior. Although signs of AF will manifest in conventional electrocardiogram (ECG) recordings, interpretation and localization of AF sources require significant clinical expertise. In this vein, electrocardiographic imaging has emerged as an important medical imaging modality that provides reconstructions of the heart's electrical activity from non-invasive multi-lead body-surface ECG and anatomical x-ray computed tomography images. In this paper, we present a nonlinear inversion model for computing this mapping to improve upon the reconstruction performance of current methods. While contemporary techniques typically determine an inverse solution by discretizing and inverting an underdetermined linear system of partial differential equations governing the relationship between voltage potentials of the heart and torso, the presented technique re-casts this problem as a task in function approximation and provides a direct parameterization of the inverse operator using a polynomial neural network. That is, the outlined nonlinear inversion technique is a generalization of contemporary reconstruction techniques which allows geometrical and material parameterizations of the forward-model to be optimized using real experimental data collected from patients suffering from AF, as to better represent the inverse operator with respect to reconstruction metrics applicable to electrophysiology. The accuracy of our model is evaluated against a dataset of real-patient recordings to demonstrate its validity, and mathematical analysis is provided to support the polynomial expansion used in our inversion model.

## I. INTRODUCTION

Mapping of electronic cardiac potentials remains an important tool in electrophysiology, particularly for diagnosing and treating various types of cardiac arrhythmias including premature ventricular contractions (PVC), ventricular tachycardia (VT), atrial flutter, and atrial fibrillation (AF). While a majority of arrhythmia cases can be identified from multi-electrode body-surface electrocardiogram (ECG) measurements, clinicians performing interventions often rely on more local electronic mappings of the cardiac tissue (e.g., using multi-probe catheters) to classify the type of arrhythmia, localize its source, and determine whether an intervention procedure such as an ablation is recommended for the patient.^1,2^ Unfortunately, the physical contact between the catheter probes and the epicardial or endocardial tissue is typically a requisite to build these descriptive 3D cardiac potential maps, forcing clinicians to perform these procedures during planned surgical interventions. From a diagnostic perspective, studying the time-resolved 3D cardiac map of a patient *prior* to surgery can improve patient outcomes by helping clinicians identify and localize dominant AF or PVC sources, determine whether an ablation procedure would be an effective treatment (e.g., for long-standing AF or when AF sources cannot be localized), and monitor patients' electrophysiology conditions over time and during regular physical activity.^3,4^ In this vein, the goal of this paper is to outline a new *noninvasive* technique that can aid in the construction of these cardiac potential maps.

This problem is known in the field as electrocardiographic imaging (ECGi) and has been studied extensively by a number of research groups.^5–7^ The core mathematical problem of ECGi can be thought of as a special case of the more-general inverse scattering problem of electromagnetics, which involves determining characteristics of an object (in this case, the potentials of the heart), based on data of how it scatters incoming radiation.

Even in linear homogeneous media, however, the problem is often ill-posed because the number of measurements is small relative to the number of unknown physical or geometric parameters required in the model. To this end, various groups have approached this problem by (1) modeling the forward electromagnetic problem using computationally efficient linear formulations, such as the boundary-element method, (2) “inverting” the transformation to represent the inverse solution, and (3) adding a regularization constraint on the solution to both mitigate the ill-posed nature of the problem and help find a unique solution.^8,9^ Although such methods have shown promise in a number of imaging applications, including ECGi, the reconstruction accuracy has (empirically) been limited by the complexity of the forward and inverse models. Said another way, the reconstruction accuracy in these applications is typically reflective of the chosen forward and inverse models' ability to capture the intricate relationship between cardiac source-potentials and body-surface measurements and is thus degraded when assumptions (e.g., of linearity, homogeneity, and source-free regions) are violated. In particular, in patient-specific applications, where it is common for a number of the materials and geometries to be estimated with high uncertainty or entirely unknown, simple forward models of linear homogeneous media and the corresponding inverse models are often only sufficient as a first-order reconstruction heuristic^10,11^ and largely insufficient for accurately reconstructing higher-order spatial and temporal harmonics.

While these simplifying assumptions in the forward model have historically been integral to the formulation of classical imaging techniques and the field of Fourier optics, in this paper, we demonstrate how a direct, non-linear parameterization of the inverse problem can lead to more accurate reconstructions of cardiac potentials from torso measurements. That is, while the majority of previous ECGi studies have focused on developing the forward and inverse maps from idealized material geometries, we instead cast the problem of inverse imaging as a task in function approximation, where the material parameters can be either postulated or entirely unknown. In line with techniques used in non-linear optics, the described technique relies on an approximation of the inverse map using a high-degree polynomial but whose gradient is bounded. The parameters of this model are found by “training” or optimization using historical data of cardiac potentials (measured using catheter probes), corresponding body-surface potentials, and their relative 3D location on the surface of real patients who underwent surgical interventions. In general, these historical data can include reconstruction parameters extracted from previous measurements of the same patient or from a database of different patients with varying torso and cardiac geometries. The idea here is to replace the classically simple but rigid geometrical models of the torso with more flexible parametrizations that can adapt to more realistic patient geometries involving multiple dielectric media, even when these are not explicitly known in the forward model.

In this vein, we note that the presented inversion technique can be used in two modes: (1) for initialization and fine-tuning of the inverse map from body-surface (BS) potentials to endocardial potentials and (2) for non-invasive electronic imaging of cardiac tissue. Mode (1) can be used, for example, to study the properties of the interstitial tissue between the torso and the endocardium (e.g., density and permittivity). However, mode (2) would be used at a later time, when such catheters are removed from the patient, and only BS potentials are available to study cardiac activity (e.g., accurate localization of the site of the origin of PVC or focal VT) and for personalized procedure planning (e.g., ablation or other surgical interventions). While current ECGi methods provide this capability in a number of ways,^12^ they have been limited in their reconstruction accuracy, limiting their effectiveness in understanding and non-invasively localizing the source of AF, PVC, and VT.^13^ To this end, the presented algorithms attempt to increase the accuracy of reconstructed endocardial potentials by incorporating a learning framework with a naturally parametrized nonlinear reconstruction model. The resulting system is capable of using an array of electrocardiogram (ECG) signals (with the corresponding electrode locations), target mesh locations, and a parametric reconstruction model (summarized as a polynomial network with variable coefficients), to produce an accurate 3D cardiac potential map.

From a practical viewpoint, we believe that our approach can improve the analysis capabilities of cardiologists and electrophysiology specialists who are interested in studying cardiac events in live patients. Compared with linear formulations, the presented approach provides enhanced spatio-temporal resolution and reconstruction accuracy, which can help in identifying, localizing, and characterizing dominant sources of AF and PVC including higher-order temporal harmonics from high-quality localized maps of cardiac potentials rather than simple projections of this information.^7,12,14^

## II. RESULTS

To evaluate the proposed approach, a set of medical data was analyzed to determine the effectiveness of the presented reconstruction technique with respect to other contemporary methods, such as formulations based on the boundary element method (BEM). The accuracy of our inverse solution is strongly dependent on the precision of historical measurements that are used to optimize our inverse operator. Due to this dependency, we identified a curated open-source data archive of high-quality *in vivo* measurements collected for physiological and medical studies of the cardiovascular system. For this experimentation, the electrical measurements were acquired with precision medical instrumentation to ensure high accuracy, and other medical imaging data were also collected to supplement the ECG data.^7^ In this section, we highlight our results through visualizations of imaging and reconstruction results, as well as the accuracy of the reconstructions.

### A. Non-invasive imaging and reconstruction of endocardial potentials

The inverse model was optimized on the set of body-surface potential measurements (BSPM) and invasive endocardial measurements that were used to reconstruct the endocardial potential map, as described in Sec. IV A. Given that both these measurements were recorded simultaneously for the given dataset, sufficient information was available to train a model to estimate this inverse function. To achieve this with the limited data source, the body surface and endocardial measurements were separated into two disjoint sets. From the original data, time samples were randomly chosen to serve as the training data. After the training data were used to optimize the inverse network, the remaining data were used to test the reconstruction accuracy. Because the time samples were chosen at random sample instances, the learned inverse model is implicitly time invariant.

**FIG. 1.**
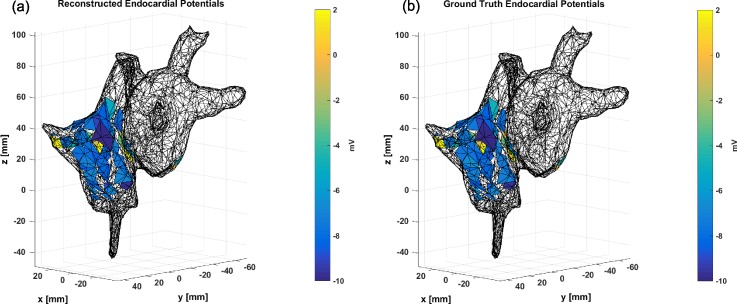
Reconstruction of the endocardial potentials (a) and measured ground truth endocardial potentials (b) on the heart.

With the catheter endocardial measurements available, we were able to compare our reconstruction estimate to a “ground truth” measurement. Furthermore, with the geometry of the atria obtained from x-ray computed tomography (XRCT) imagery, the reconstructed endocardial potentials were mapped to spatial positions on the endocardial surface for an accurate visualization of the voltage spatial profile as in Fig. 1. The reconstructed potential is compared to the ground truth measured potentials again, and close correspondence between the two mappings is clear. The spatial-potential information here is a useful tool to determine and isolate concentrations of electrical activity in the atria, to further aid in the noninvasive study of the electrophysiology of the cardiovascular system.

**FIG. 2.**
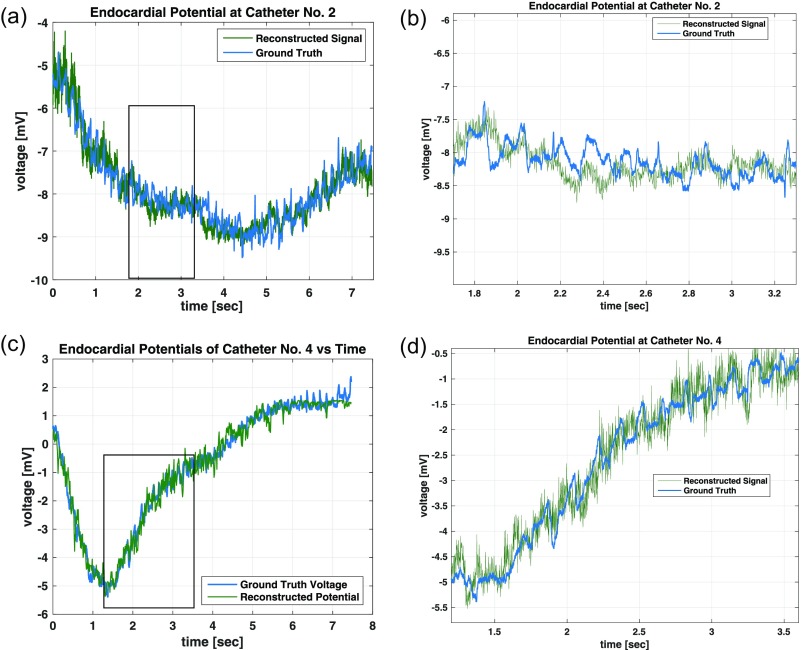
Shown here are small-window time-domain reconstructions of endocardial potentials from body-surface potentials via the presented nonlinear imaging technique.

**FIG. 3.**
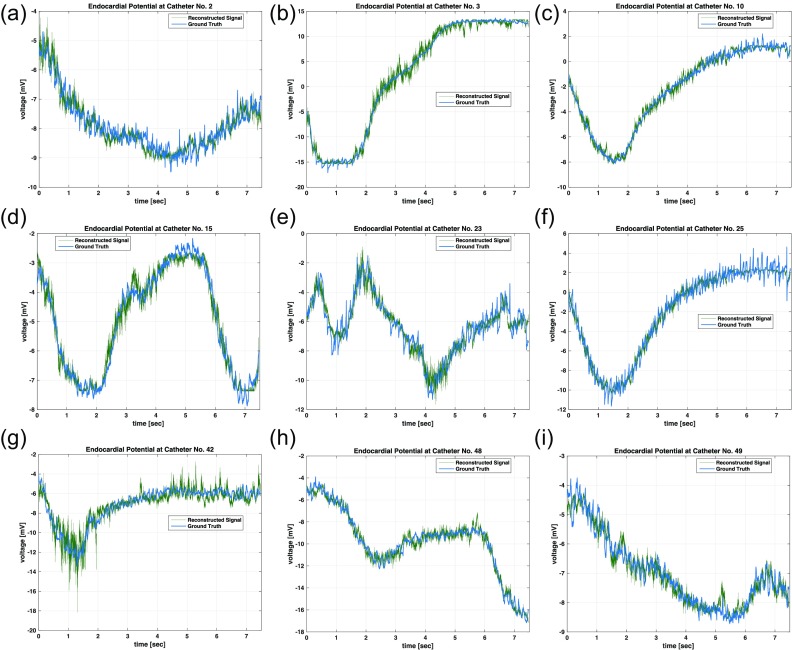
The presented nonlinear imaging technique produces high-quality time-domain reconstructions of endocardial potentials at various catheter positions.

Two important factors under consideration when interpreting inverse mappings of BSPM to endocardial potentials are preservation of temporal features such as harmonic content and waveform shape and also the smoothness of the spatial profile of the voltage pattern of the heart's surface. A temporal plot of the endocardial reconstructed voltage signal at multiple points on the heart's surface can be seen in Fig. 2, which can be used to develop intuition into how the inversion model reconstructs the signal. The reconstructed signal accurately tracks slowly changing features in ground truth signals over the 7-s measurement interval. The low-frequency content indicates accurate preservation of lower order harmonics by the inversion model, which is an important feature for the spatio-temporal understanding of the physiology of AF. Furthermore, the relative magnitude and scale are also preserved in the reconstruction without the need for estimated scaling parameters, so as to provide interpretable results for users not familiar with the specific inversion method. The reconstructed signals in Fig. 3 are also representative of this qualitative analysis.

### B. Accuracy of reconstructed endocardial voltage potentials

The performance of the reconstruction model was evaluated using several statistics. The mean absolute error, mean relative error, and their standard deviation are displayed in Table I. The average absolute error over the entire test dataset was found to be 0.327 mV, which yielded a 12.47% average relative error with respect to the recorded data. Additionally, the training results are of similar accuracy to the reconstruction results, indicating that the model is robust to new test data and has not been over-fitted to the training data, which is an important quality for any inversion approach.

**TABLE I.** Reconstruction performance.

	Absolute error (mV)	Relative error (%)
Training results	0.310 ± 0.321	12.07
Reconstruction results	0.327 ± 0.221	12.47
Normalized reconstruction results	0.327 ± 0.146	12.43
Normalized BEM results^7^	…	35.8

These results indicate that a high degree of confidence may be placed in the reconstruction of the endocardial potential distribution using the proposed methodology. As a comparison in Table I, we list the accuracy of the inverse computed BEM results reported in Ref. 7. Here, it should be noted that relative accuracy was reported for the normalized voltage signals of the endocardium due to numerical scaling issues when computing BEM inverse solutions. This metric describes how well the inversion procedure preserves the overall shape of the endocardial waveform, if not the true scale. Here, our proposed reconstruction method noticeably outperforms the BEM solution on average over the entire dataset.

**FIG. 4.**
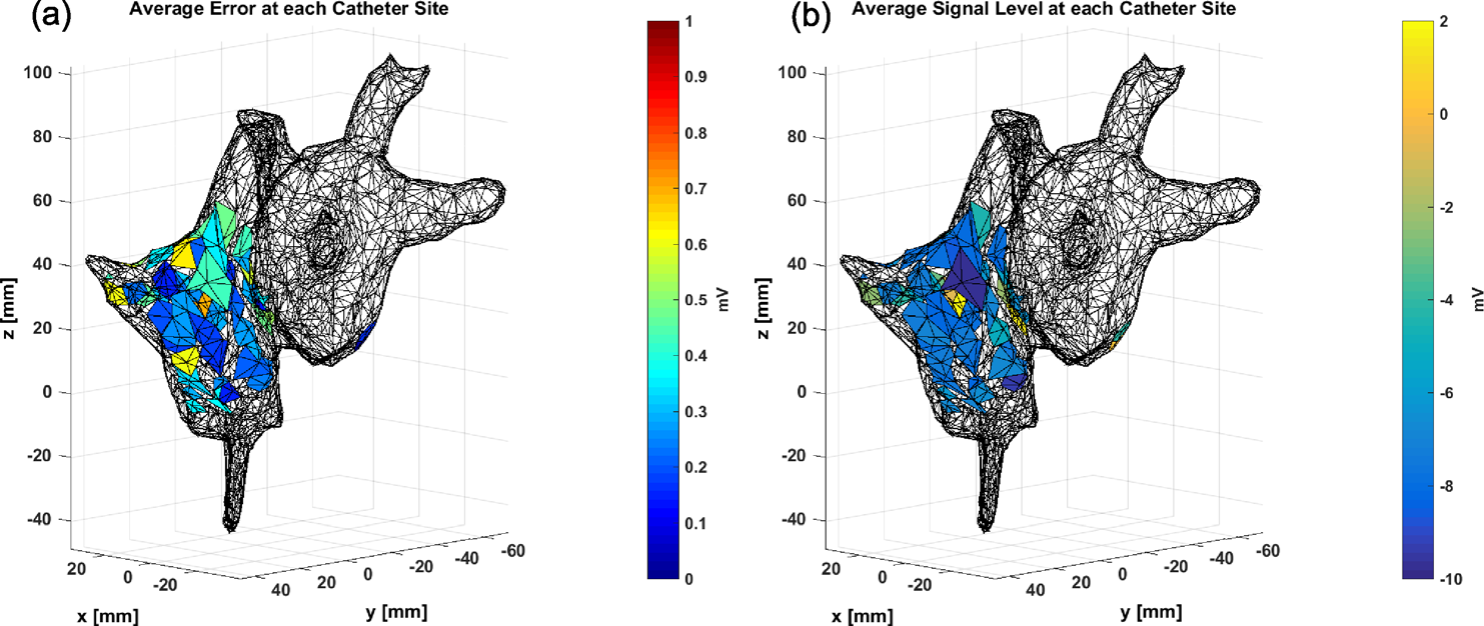
The average reconstruction error (a) is small over the spatial area where ground truth data were collected, with a maximum absolute error less than 0.70 mV. This figure is compared in the context of the average signal level (b), which varies by as much as 12 mV over the different catheter sites.

The average reconstruction error for each sensor was also averaged across the entire time series, as can be seen in Fig. 4. Here, the average error does not exceed 0.7 mV over the various sensor positions, indicating that the reconstruction model does not suffer from any spatial bias. Therefore, at any point in time, given body surface potential measurements, this model could be used to accurately reconstruct endocardial voltage potentials of the heart to within a prescribed tolerance.

## III. DISCUSSION

The results summarized here demonstrate the potential of a flexible inverse model that can be optimized with real, high-quality measured data. The optimization can help mitigate the effects of unknown physical quantities and parameters typically associated with inverse problems. The accuracy of the reconstructed cardiac potentials in the spatial-temporal domain is indicative of the validity of our optimized inverse model in comparison to other techniques. This is an important finding given that our inversion method differs from the direct inversion of the forward model problem and suggests that improvements can be made on the simplified physical models that are typically used.

**FIG. 5.**
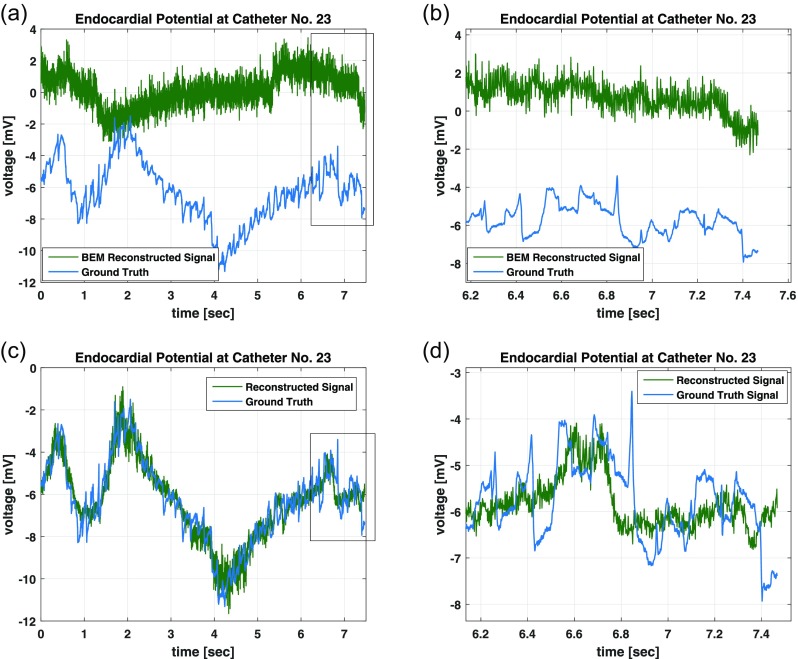
Time-domain reconstructions using a standard BEM method can produce significant error in the voltage signal (a) and (b). The presented polynomial reconstruction technique can both recover a significant portion of the missing large-signal information (c) and still exhibit some temporal inaccuracies in the small-signal reconstruction due to the inherent time-invariant nature of the model (d). (a) Catheter No. 8 Time Reconstruction (b), Catheter No. 8 Time Reconstruction Zoomed (c), Catheter No. 8 Time Reconstruction, and (d) Catheter No. 8 Time Reconstruction Zoomed.

Boundary-element method (BEM) solutions to the inverse problem have shown some success in the reconstruction of relatively smooth cardiac potential maps. However, if the potential map is highly varying over time, the regularization introduced in the inversion will tend to smooth out complex features that are exhibited during AF events and thus decrease accuracy. Given that our inversion model was optimized over BSPMs and endocardial data acquired from a patient being treated for AF, these complex patterns in the potential maps are properly reproduced by our model [Fig. 5(c)].

While our inverse model exhibited the high accuracy in voltage potentials in the time domain, there were some observed limitations in the spectral accuracy of the reconstruction. Specifically, low amplitude, high frequency transients were difficult to estimate with the model. This type of feature was observed in the original data, as can be seen in Fig. 5(d) along with our reconstruction result. This limitation is due to the small error contributed to the overall objective function measured over the entire dataset. The optimization will instead reconstruct a mean constant value during these events which still yields a low-average error for the entire duration of the signal. In this respect, the proper fidelity of the high frequency content of the endocardial signals continues to be a limitation faced by other inverse solutions.^15^

The acceptance of a particular solution to inverse solutions in ECGi is still contested in the field.^10^ The debate focuses on identifying and defining the optimal features in cardiac potential mappings that best serve clinical and diagnostic goals. In general, spatio-temporal accuracy is almost universally regarded as critical for successful inversion of BSPMs, as invasive techniques used in ablation procedures mainly rely on surgeons manually probing the endocardium and/or epicardium in real-time to identify sources of AF. Although our approach optimized error in the temporal potential maps, other waveform constraints could easily be incorporated in our objective functions to help detect features of interest. This could be achieved, for example, by including a generalized Tikhonov regularization in addition to the Sobolev-norm regularization that was used, e.g., via a non-uniformly weighted-norm or band-pass filter.

Finally, we note that while the presented methods have demonstrated quantitative improvements in the time-domain-independent reconstruction of cardiac potential maps from BS potentials, the ultimate utility of this application depends on the clinical and diagnostic needs of electrophysiologists who would rely on such information to refine patient prognosis. In particular, the presented approach to non-invasive cardiac mapping provides a promising outlook for the use of non-invasive ECGi to monitor and study cardiac disease because it offers a flexible learning-based framework that leverages previous measurements to refine features of cardiac reconstructions that hold diagnostic value.

## IV. METHODS

The authors state that an ethics approval is not required.

### A. Experimentation and data collection

The experimental data in this paper were provided through the Consortium for Electro-cardiographic Imaging (CEI), a group of engineers, scientists, and clinicians who develop clinically and physiologically meaningful tools for simulation, modeling, statistical, and comparison studies of electrocardiographic imaging. The experiments were conducted at the Hospital General Universitario Gregorio Maranon in Madrid, Spain, in collaboration with the Universitat Politecnica de Valencia in Valencia, Spain. In this paper, we examine electrical measurements of one male patient (aged between 40 and 50 years old), who was admitted for drug-refractory paroxysmal atrial fibrillation. The voltage measurements were composed of both body surface (BS) potential mappings and endocardial surface potentials. The body surface potential maps were obtained on the surface of the patient's torso with a custom made electrocardiographic vest, and the endocardial potentials were measured through an electrical catheter probe in contact with the surface of the atria.^7,14^

Non-invasive reconstruction of the potential maps on the atria's surface requires mapping of the body surface potential across the subject. To measure the body surface potential maps (BSPMs), multiple electrocardiograms (ECGs) were measured at discrete positions on the patient's torso. A total of 54 ECGs were measured with body surface leads placed on the torso surface and electrically referenced to the Wilson Central Terminal (WCT). To determine the locations of the BS leads, a 3D model of the patient was constructed from multiple optical images captured prior to the data collection. Additionally, the surface geometry of the patient's torso and atria was obtained from pre-scanned XRCT images with a resolution of 5 mm. These CT images were then co-registered to the optically generated 3D models to ensure that the BS lead locations, torso surface, and atria surface geometries were also referenced to the same coordinate system.^16,17^ It can be noted that the proposed reconstruction method does not explicitly require high accuracy positional information.

**FIG. 6.**
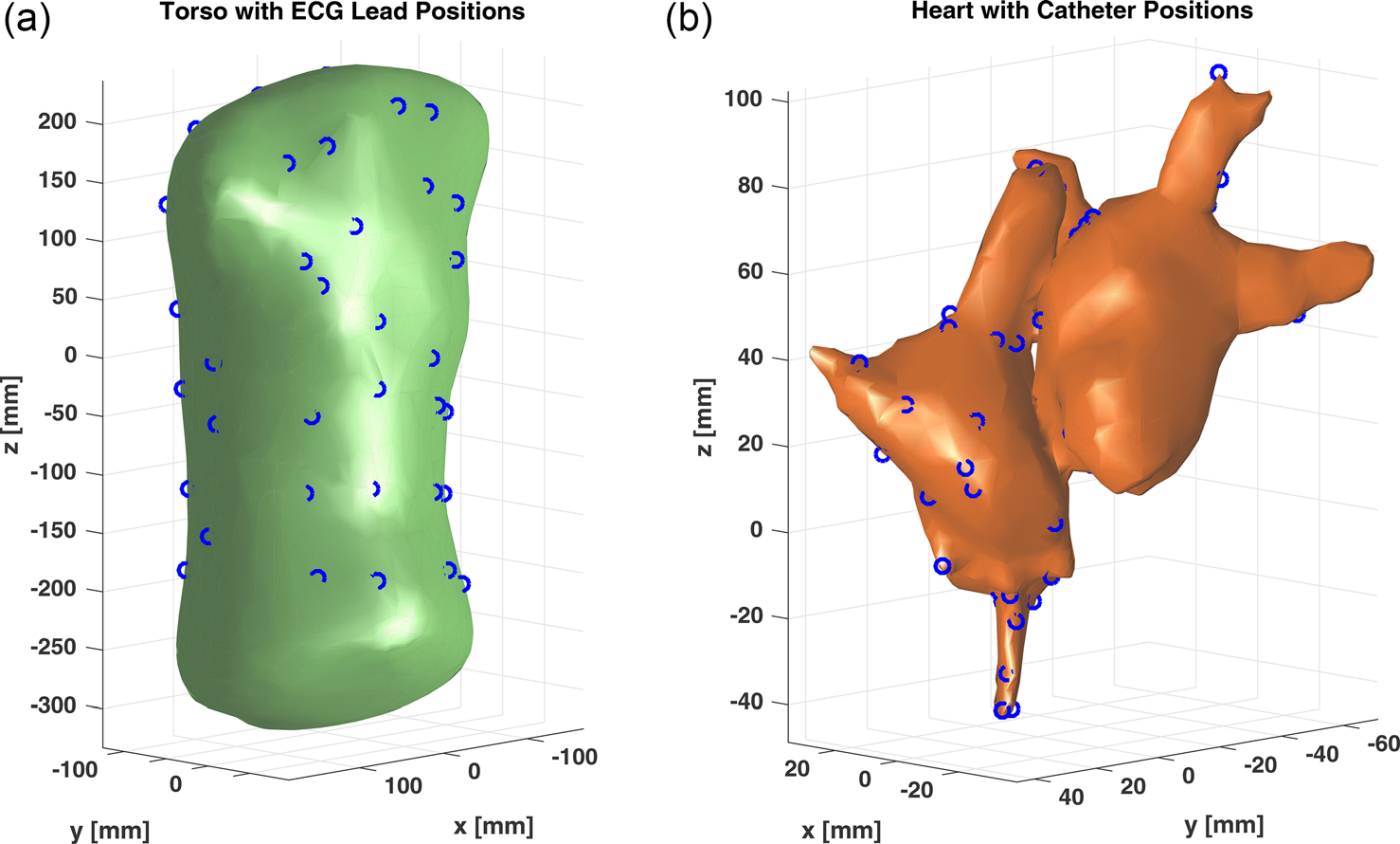
The surface mesh of the torso (a) and heart (b) is shown here, along with the corresponding locations of the electrical leads.

Similar to the BSPMs, multiple endocardial potentials were also recorded internally at different spatial positions. These electrical signals were acquired with a 64-pole basket catheter (Constellation, Boston Scientific, Natick, MA), surgically placed in contact with the surface of the atria. The position of the catheter leads was recorded through the catheter's internal body navigation system. The positional accuracy of the leads was reported to be < 10 mm with respect to the geometry of the atria's surface. A rendering of the torso's surface mesh and heart surface mesh along with the locations of the electrical leads can be seen in Fig. 6. The ECG recordings for both the endocardial leads and BS leads were acquired simultaneously at a fixed sampling rate of 2035.5 Hz. A total length of 7.4 s of data was recorded from all channels, which was used to help construct our inversion model as discussed in more detail in Sec. IV B.

### B. Inverse function modelling

As mentioned, conventional approaches to ECGi have considered inversion techniques based on simplifications of the more general inverse scattering problem, e.g., by assuming linearity, the number and geometry of dielectrics, and the distribution of excitation sources within the endocardium. To a large extent, these approaches to the linear inverse problem can be summarized as modeling the relationship between the potentials on the atrial surface *U_A_* and potentials on the torso *U_T_* as

$$U_{T}=M\cdot U_{A},$$ (1)

where *M* represents a linear operator that can be constructed in a number of ways from the assumed geometry, material parameters (e.g., permittivities and permeabilities), governing equations of the medium (e.g., Maxwell's equations and Coulomb's law), and the chosen discretization and associated approximation scheme (e.g., diffeo-integral equation formulation as boundary-elements, finite-volumes, or finite-differences). While in general *M* represents an infinite-dimensional operator, in practice, it is realized as a finite-dimensional operator *M_k_* representing a subset of the corresponding rows of *M*. However, for clarity in our presentation, we will use *M* to refer to *M_k_*.

Of course, to a large extent, the materials and geometries of the torso can be estimated with considerable accuracy, using noninvasive techniques such as x-ray computed tomography (XRCT) and magnetic resonance imaging (MRI) techniques, as well as historical anatomical measurements from cadavers. In fact, these are often used to initialize discretized models of *M* used in the forward formulation, even though the condition-number of this matrix is typically large, resulting in large changes to the unknown *U_A_* with respect to relatively small perturbations of the known *U_T_*. This property is reflective of the inherent ill-posed nature of the inverse problem and is typically handled by addition of a regularization parameter that is computed as a part of the reconstruction. For example, in medical imaging, the zero-order Tikhonov method, which is a type of $L^{2}$ regularization, is used as

$$U_{A}={\left(M^{T}\cdot M+\lambda I\right)}^{-1}M^{T}U_{T},$$ (2)

where the regularization parameter *λ* is chosen heuristically during the image formation process, and the choice *λ* = 0 corresponds to the usual least-squares solution to a discrete form of Eq. (1).

In this work, we consider a simple modification to this formulation that allows us to optimize and reduce uncertainty in the material parameters and geometries that arise in discrete matrix *M* based on historical data of patients, thereby resulting in a lower overall reconstruction error. In short, we consider the replacement of the Moore-Penrose operator used in the least-squares solution, by a power-series expansion. For example, if *A *=* M* is a finite-dimensional square invertible matrix, its inverse can be written down exactly as

$$p(A)=A^{n}+c_{n-1}A^{n-1}+\cdots +c_{1}A+{\left(-1\right)}^{n}\det(A)I_{n},$$ (3)

where the coefficients *c_i_* are given by elementary symmetric polynomials of the eigenvalues of *A*, and these polynomials can be re-written using Newton identities in terms of the power sum symmetric polynomials of the eigenvalues $s_{k}=\sum_{i=1}^{n}\lambda_{i}^{k}=\text{tr}(A^{k})$ as

$$\begin{array}{c} A^{-1}=\frac{{\left(-1\right)}^{n-1}}{\det\;A}(A^{n-1}+c_{n-1}A^{n-2}+\cdots +c_{1}I_{n})\\ =\frac{1}{\det\;A}\sum_{k=0}^{n-1}{\left(-1\right)}^{n+k-1}\frac{A^{n-k-1}}{k!}B_{k}(s_{1},-1!s_{2},2!s_{3},\ldots ,{\left(-1\right)}^{k-1}(k-1)!s_{k}), \end{array}$$ (4)

where *B_k_* represent the Bell polynomials of order-*k* and *n* is the dimension of *A*.

Besides allowing for better numerical control over the inverse, using this expansion also enables us to directly optimize parametrizations of the matrix *M*, i.e., by finding more suitable dielectric parameters, attenuation, or absorption coefficients, which minimize the maximum residual error between ground truth measurements of *U_A_* and transformations of the input *U_T_*, denoted as ${\hat{U}}_{A}$. In practice, we truncate the series at a smaller number *p *<* n* for run-time efficiency, even though a slightly higher number is typically used during the optimization phase for optimal results.

### C. Polynomial neural network

We generalize this scheme to scenarios where *M* is slightly non-square,^18,19^ by introducing additional weighting parameters that are used as a surrogate for the determinant computation (i.e., scaling), and empirically mitigate the effects of series truncation. In particular, by noticing that the expansion in Eq. (4) represents an (*n* – 1)-th order polynomial expression in *A*, we can re-interpret the reconstruction algorithm as a high-dimensional polynomial approximation algorithm or a neural network.^20,21^ That is, we can generalize Eq. (4) as

$$A^{-1}\approx \sum_{k=0}^{n-1}\omega_{k}(A)\cdot A^{n-k-1},$$ (5)

$$\approx \sum_{k=0}^{p}{\hat{\omega }}_{k}(A)\cdot A^{k},$$ (6)

where we have intentionally absorbed both the geometry dependent and independent coefficients of the summation into the parameters ${\hat{\omega }}_{k}$ and truncated the series to a summation of the first *p* powers of *A*. The benefit of this formulation is that it can be easily expanded to mimic even more general polynomial approximation algorithms. For example, in our experiments, we naturally expanded this formulation [Eq. (6)] by composition as

$${\hat{U}}_{A}=\sum_{k=0}^{p}\omega_{k}⊙{\left(M\cdot U_{T}\right)}^{k},$$ (7)

where ⊙ represents the element-wise Schur-product and ${\hat{U}}_{A}$ is the reconstructed $R^{m\times 1}$ potential map. We allow $\omega_{k}\in R^{m}$ for $M\in R^{m\times n}$, and we empirically chose *p *=* *3 in our experiments. We note that when $\omega_{k}=0\;\forall \;k\neq 0$ and $\omega_{0}=e_{m}$, the reconstruction algorithm is exactly equivalent to the linear case. From a practical point of view, we believe that this initialization yields desirable improvements in the overall accuracy even when trained with only simple descent strategies, as described in the following.

### D. Regularized gradient descent with line search

As mentioned in our experiments, the parameters of *M* were found via optimization using instantaneously corresponding pairs of body-surface (torso) potential *U_T_* and endocardial potential *U_A_* which were measured *in vivo*. Starting from an initial estimate of the parameters (based on simple forward/inverse models of the problem), gradient descent with line search was used to optimize our inverse model with respect to the available patient training data. That is, the “optimal” parameters of *M* can be found by minimizing the objective

$$\hat{M}=\underset{M}{\text{arg}\;\text{min}}E=\underset{M}{\text{arg}\;\text{min}}||{\hat{U}}_{A}-U_{A}|{|}_{2}^{2},$$ (8)

where ${\hat{U}}_{A}$ was computed from Eq. (7) and *U*_*A*_ represents the ground truth atrial voltage signal. To mitigate the effects of limited data (e.g., small aperture size), discretization, and modeling errors during reconstruction, we augment this objective with a Sobolev-norm regularization term as

$$\hat{M}=\underset{M}{\text{arg}\;\text{min}}E=\underset{M}{\text{arg}\;\text{min}}||{\hat{U}}_{A}-U_{A}|{|}_{2}^{2}+\lambda ||\nabla_{U_{T}}{\hat{U}}_{A}|{|}_{s}^{s},$$ (9)

which provides a bound on the roughness of the reconstruction algorithm with respect to the input data. The Sobolev-norm is known in approximation theory to be a natural surrogate for conventional $L^{2}$ regularization such as total-variation (TV) or Tikhonov regularization, which are often sensitive to the choice of *λ.*^22,23^ Instead, weighting the objective function by the Sobolev-norm of the differentiable inverse map allows practitioners to analytically tune the sensitivity of the reconstruction algorithm to the *input data* rather than using perceptual image-quality metrics on the output reconstruction (e.g., graphical L-curve method).^24,25^ It is worth noting that in this formulation, regularization is applied only during the “training phase” and *not* during reconstruction.

In our experiments, different values of *M* were found and evaluated via the update strategy

$$M_{t+1}=M_{t}+\gamma_{t}\nabla_{M}E,$$ (10)

where $\nabla_{M}E$ represents the normalized direction of the gradient of the objective function with respect to the parameters of *M*, and $\gamma_{t}\in [-1,1]$ is the step-size that was selected to minimize the objective at training iteration *t*. In practice, *γ_t_* is selected from a discrete set of *r* trials, by applying updates along the gradient direction (with different magnitudes), evaluating the objective at these various points as $E_{t,r}(M)$, and selecting the update that yields the lowest error.

### E. Measurement of errors

In the described experiments, we used the raw BS and endocardial measurements available in the described dataset, with little to no pre- or post-processing. The reported absolute and relative errors were computed directly from the output of the polynomial network at each discrete time-point *k* and averaged as follows:

$$e[k]=|U[k]-\hat{U}[k]|,$$ (11)

$$\text{Mean}\;\text{Absolute}\;\text{Error}\;[\text{mV}]=\frac{1}{T}\sum_{k}^{T}e[k],$$ (12)

$$r[k]=\frac{|U[k]-\hat{U}[k]{|}^{2}}{U[k]}\;\forall \;U[k]\neq 0,$$ (13)

$$\text{Mean}\;\text{Relative}\;\text{Error}\;[\%]=\frac{1}{T\prime }\sum_{k}^{T\prime }r[k]\;\forall \;k\in T\prime \;s.t.\;U[k]\neq 0.$$ (14)

In particular, we note some measurements (a total of 6 time-points over the entire dataset) were excluded from the relative error computation (i.e., when the recorded endocardial potential value was effectively 0) for interpretability. Measuring both the absolute and maximum error ameliorates the missing data in these cases.
